# Targeting MAD2 modulates stemness and tumorigenesis in human Gastric Cancer cell lines

**DOI:** 10.7150/thno.49270

**Published:** 2020-07-25

**Authors:** Natalia Pajuelo-Lozano, Sonia Alcalá, Bruno Sainz, Rosario Perona, Isabel Sanchez-Perez

**Affiliations:** 1 Dpto. Bioquímica. Fac. Medicina. UAM, Madrid, Spain.; 2 Instituto de Investigaciones Biomédicas “Alberto Sols” CSIC-UAM, Madrid, Spain.; 3 Cancer Stem Cell and Fibroinflammatory Microenvironment Group, Chronic Diseases and Cancer Area 3 - Instituto Ramón y Cajal de Investigación Sanitaria (IRYCIS), Madrid, Spain.; 4 Unidad Asociada de Biomedicina UCLM-CSIC.; 5 CIBER for Rare Diseases (CIBERER); Valencia, Spain.

**Keywords:** gastric cancer *stem*-like cells, MAD2, EMT, MMPs, tumorigenesis

## Abstract

**Rationale:** Gastric cancer (GC) is a solid tumor that contains subpopulations of cancer *stem* cells (CSCs), which are considered drivers of tumor initiation and metastasis; responsible for therapeutic resistance; and promoters of tumor relapse. The balance between symmetric and asymmetric division is crucial for *stem* cell maintenance. The objective of this study is to evaluate the role of MAD2, a key protein for proper mitotic checkpoint activity, in the tumorigenesis of GC.

**Methods:** Gastric cancer *stem* cells (GCSCs) were obtained from MKN45, SNU638 and ST2957 cell lines. Pluripotency and *stemness* markers were evaluated by RT-qPCR and autofluorescence and membrane markers by flow cytometry. Relevant signal transduction pathways were studied by WB. We analysed cell cycle progression, migration and invasion after modulation of MAD2 activity or protein expression levels in these *in vitro* models. *In vivo* assays were performed in a nude mouse subcutaneous xenograft model.

**Results:** We found that *NANOG*, CXCR4 and autofluorescence are common and consistent markers for the GCSCs analysed, with other markers showing more variability. The three main signalling pathways (Wnt/β-catenin; Hedgehog and Notch) were activated in GCSCs. Downregulation of MAD2 in MKN45^CSCs^ decreased the expression of markers CXCR4, CD133, CD90, *LGR5* and *VIM*, without affecting cell cycle profile or therapy resistance. Moreover, migration, invasion and tumor growth were clearly reduced, and accordingly, we found that metalloprotease expression decreased. These results were accompanied by a reduction in the levels of transcription factors related with epithelial-to-mesenchymal transition.

**Conclusions:** We can conclude that MAD2 is important for GCSCs *stemness* and its downregulation in MKN45^CSCs^ plays a central role in GC tumorigenesis, likely through CXCR4-*SNAI2*-MMP1. Thus, its potential use in the clinical setting should be studied as its functions appear to extend beyond mitosis.

## Introduction

Gastric cancer (GC) is the fifth most frequent neoplasm and the third deadliest cancer in the world (GLOBOCAN 2018). Treatment strategies for localized tumors include surgery followed by chemo- or radiotherapy. Despite the development and identification in recent years of novel anticancer agents and treatment alternatives, a high percentage of GC patients eventually relapse, resulting in an overall 5 year survival rate of only 20-30% 1. In GC, drug resistance, metastasis and recurrence may be largely due to the existence of GC *stem*-like cells (GCSCs) within the tumor 2, 3. CSCs possess the ability to self-renew and differentiate into multiple lineages, and they have been described as the main contributors in tumor aggressiveness, metastasis, chemotherapy resistance and relapse 4. CSCs express specific markers, which depend on the type of tumor and the specific subpopulation of cells from which they originate. Several markers have been described for GCSCs, including CD44, CD90, CD133, EpCAM, ALDH1, CXCR4 and LGR5 5. The level of expression of these markers depends on their origin and also on the properties of the primary tumor, which makes it mandatory to perform specific studies to thoroughly characterize the GCSC subpopulation within each tumor or cell line 6. In general, CSCs show activation of the major signaling pathways: Hedgehog, Wnt/β-catenin and Notch, which are implicated in the regulation of epithelial-to-mesenchymal plasticity (EMP), one of the main characteristics leading to the described aggressiveness of these cells 7.

A CSC can follow one of two paths: differentiation or self-renewal. If it divides asymmetrically, the result will be one CSC and one specialized differentiated cell. However, the alternate self-renewal path results in two identical CSCs. Thus, like *stem* cells, CSCs undergo hierarchical branched division 8. During this process, cell cycle checkpoints play a key role, especially the mitotic checkpoint. The spindle assembly checkpoint (SAC) is a complex of proteins that includes MAD1, MAD2, BUB1, BUBR1, BUB3, and MPS1, which controls proper spindle formation and orientation ensuring that every chromosome kinetochore is correctly attached to centrosome microtubules. MAD2 participates in the mitotic checkpoint complex (MCC), which can bind and inhibit the anaphase promoting complex (APC/C). When all kinetochores have correctly attached, the SAC turns off and the MCC disassembles, thus freeing CDC20 for APC/C activation. The APC/C-CDC20 complex targets securine and cyclin B1 for degradation resulting in sister chromatid separation and mitosis exit, respectively. The cleavage plane also establishes the partition of cellular contents, including cell-fate determinants. This is critical for *stem* cell self-renewal and differentiation 9, 10. The role of several checkpoint proteins has been studied in cancer, like MAD2 and BUBR1, and the levels of these proteins have been associated with tumorigenesis or clinical prognosis 11-13.

GC presents a large degree of inter- and intratumoral heterogeneity, which can have both genetic and non-genetic bases. Chromosomal instability (CIN) can be responsible for high intratumoral genetic heterogeneity. The non-genetic heterogeneity (i.e epigenetics and microRNAs) is the heterogeneity that arises due to EMP, to the ability of cells to maintain their plasticity and transit between epithelial-mesenchymal transition (EMT) and mesenchymal-epithelial transition (MET) 14. These cell transitions allow cells to migrate away from the primary tumor and thus invade secondary sites. EMT is a transcriptionally-mediated process and is associated with cell morphological changes that result in enhanced cellular migration and invasion, the latter of which is facilitated by degradation and remodeling of the extracellular matrix, the sum of which ultimately leads to the successful colonization of cancer cells at secondary sites 6. In this process of matrix remodeling, matrix metalloproteinases (MMPs) play an essential role degrading the basement membrane and extracellular matrix. There is evidence that some MMPs are implicated in EMT induction, such as MMP3, which directly regulates E-Cadherin and also participates in the regulation of the Wnt pathway 15. Levels of MMPs, such as MMP1, 2 and 9, have been found to be increased in GC, and their overexpression has been shown to be associated with tumor invasion and metastasis 16. EMT is tightly correlated with the activation of EMT transcription factors, such as ZEB1, SNAI1 (SNAIL), SNAI2 (SLUG) and TWIST 17. However, during EMT progression, cells can exhibit a hybrid epithelial/mesenchymal (E/M) phenotype, in which cells will co-express epithelial and mesenchymal markers. In fact, *stemness* also associates with cells that adopt the hybrid E/M state 18, inducing tumor cells to develop *stem* cell characteristics, which promote cells to invade surrounding tissues and contribute to therapeutic resistance 19.

We previously described that MAD2 is overexpressed in several GC cell lines 13; however, the specific role of MAD2 in tumorigenesis remains controversial. Since the majority of recent work suggest that models based on CSCs are biologically more relevant, our aim was to analyze the role of MAD2 in GCSCs. We show that MAD2 is involved in the regulation of different *stem*-associated properties of the MKN45 cell line, suggesting an array of new potential functions for this protein, all of which we aim to study here in detail to elucidate possible applications for the clinical setting.

## Materials and Methods

### Cell lines and chemicals

The human GC adenocarcinoma cell lines MKN45, SNU1, and SNU638 (poorly differentiated; DSMZ: Deutsche Sammlung von Mikroorganismen und Zellkulturen GmbH), ST2957 (lymph node metastases), and AGS (primary tumor; ATCC/LGC Standards, Spain) were cultured in RPMI 1640 (Sigma), DMEM (Gibco) and HAM's F-12 + AA's (Gibco), respectively, according to the specifications of the manufacturer´s datasheet and supplemented with 10% FBS, 2 mM L-Glutamine, Fungizone 1× and 0.07% Gentamicin. Cultures were maintained at 37 ºC, 5% CO_2_ and 95% humidity. Mycoplasma contamination is routinely tested in our laboratory.

Cisplatin (CDDP) was kindly donated from Ferrer FARMA, Bleomycin (BLM) acquired from Calbiochem and Paclitaxel (PXL) and Puromycin purchased from Sigma-Aldrich. MTS was acquired from Promega and M2i-1 inhibitor was purchased from Cayman Chemical.

### Tumor sphere assay

GC cells were cultured in DMEM/F12 (Invitrogen) supplemented with B-27 (10889038, Gibco), 2 mM L-Glutamine, Fungizone 1×, 0.07% Gentamicin, 20 ng/mL EGF and 20 ng/mL bFGF. These cells were seeded in ultra-low-attachment 6-well plates (3×10^3^ cells/3 mL in each well) and maintained in a humidified incubator (5% CO_2_ at 37 °C). Fresh medium was added to the culture every few days, and the culture was analyzed daily for tumor sphere formation (up to the seventh day). Representative images were captured using a Nikon Eclipse TS100 microscope. For these experiments, cells were grown in ULA Flasks or plates for 5-6 days, and for serial passaging, spheres were harvested using a 40 µm cell strainer, dissociated to single cells with trypsin, and re-grown in the same conditions for 5-6 days (in general, secondary spheres were used for all experiments).

### RT-qPCR

Total cellular RNA was extracted using Tri-Reagent (Life Technologies), following the manufacturer's instructions. One microgram of total RNA was primed with poly-T and cDNA synthesized with M-MLV reverse transcriptase following the manufacturer's instructions (Promega). Target genes were amplified using the SYBR Green polymerase chain reaction assay, using the specific primer sets listed in the table below:

IL-8 expression was measured (Hs00174103_m1) using a TaqMan® Universal PCR Master Mix (Applied Biosystems-Thermo Fisher, P/N 4304437, Foster City, CA), and GAPDH (Hs99999905_m1). Thermal cycling of the qPCR reaction was initiated with a denaturation step at 95 °C for 10 min, and consisted of 40 cycles (denaturation at 95 °C for 15 sec, annealing at 60 °C for 30 sec, and elongation at 75 ºC for 30 sec). PCR amplifications were carried out in a StepOne Real‐time PCR System (Applied Biosystems, 4376357). Relative mRNA levels were calculated by the delta-Ct method (2-ΔΔCt), where each 1-Ct difference equals a 2 fold change in transcript abundance, using GAPDH as an endogenous reference. ΔΔCT represents the difference between the mean ΔCT value of the cells tested and the mean ΔCT value of the calibrator, both calculated for the same PCR run.

### Western Blotting

Total protein extracts were obtained using the previously described lysis buffer 20. Twenty μg of protein per sample were loaded in 8%, 10% or 15% SDS-PAGE polyacrylamide gels, and then transferred onto PVDF membranes, followed by immunodetection using appropriate antibodies. Antibodies against the following proteins were used: SOX2 (sc-365823; 1:1000), NANOG (sc-293121; 1:1000), MAD2 (sc-28261; 1:1000), BUBR1 (sc-47744; 1:1000), Cyclin B1 (sc-166757; 1:1000) and β-ACTIN (sc-47778; 1:1000) were purchased from Santa Cruz Biotechnology; GLI1 (#3538 1:1000), NOTCH1 (#3608; 1:1000), Cyclin A2 (#4656; 1:1000), Cyclin D1 (#2926; 1:1000), phospho-CDK1^Tyr15^ (#9111; 1:2000), CDK1 (#9112; 1:1000) and phospho-Histone H3^Ser10^ (#3377; 1:1000) were all purchased from Cell Signaling Technology; Antibody to β-catenin (610154; 1:1000) was acquired from BD Transduction Laboratories and finally, α-Tubulin (1:10,000) from Sigma. Secondary antibodies conjugated with horseradish peroxidase were purchased from BioRad, and chemiluminescence detection was performed using ECL (Santa Cruz Biotechnology).

### Flow cytometry

Cells were analyzed with a 4-laser Attune NxT Acoustic Cytometer (Thermo Fisher Scientific). Samples were resuspended in FLOW buffer (1x PBS; 3 mM EDTA (v/v); 3% FBS (v/v)), and the following fluorescently tagged antibodies were used to label cells for 30 minutes at 4°C: mouse monoclonal anti-human CXCR4-PE (5:50, REA649; 130-117-354, Miltenyi Biotec), mouse monoclonal anti-human CD44-PE (1:50; 555479, BD Pharmingen), mouse monoclonal anti-human CD90-APC (2.5:50, 5E10; A15726, Life Technologies) and mouse monoclonal anti-human CD133-PEVio770 (1:50, AC133; 130-113-110, Miltenyi Biotec). Autofluorescent cells were excited with blue laser 488 nm and selected as the intersection with the filters 530/40 and 580/30 as previously described 21. DAPI was used to identify and exclude dead cells, and data were then analyzed using FlowJo v9.3 software (Tree Star Inc., Ashland, OR).

### AldeRed ALDH detection assay

The AldeRed with 588-A ALDH Detection Assay (SCR150, Millipore) was used, following the manufacturer's instructions. Briefly, 2×10^5^ cells were resuspended in AldeRed assay buffer containing the AldeRed 588-A substrate. The cell suspension was split into two fractions, one half of which served as a control, and was transferred to a new tube containing the specific ALDH1 inhibitor diethylamino-benzaldehyde (DEAB). Cells were then incubated for 45 min at 37°C in complete darkness. Next, cells were centrifuged (300 g, 5 min), their supernatants discarded, and cell pellets resuspended in 500 μL of cold AldeRed assay buffer. Samples were stored on ice and darkness prior to flow cytometry analysis (4-laser Attune NxT Acoustic Cytometer; Thermo Fisher Scientific). DAPI was used to mark and exclude dead cells, and data was analyzed using FlowJo v9.3 software (Tree Star Inc., Ashland, OR).

### Viral transduction of cells

Viral particles were generated according to the manufacturer's instructions using GIPZ Lentiviral shRNA for *MAD2L1* (Thermo Scientific Open Biosystems). Briefly, 4.5×10^6^ HEK293 cells/plate in DMEM medium were transfected using lipofectamine 2000 (Invitrogen) with 15 μg of shMAD2L1 or non-silencing shRNA, 7 μg of envelope plasmid (VSV-G) and 7 μg of Helper plasmid (pCD/NL-BH). Supernatants were recovered 48 h and 72 h after transfection, filtered and frozen in small aliquots at -80°C until use. Infections were performed using 5×10^5^ of indicated cells per well in a 6-well plate with 1 mL of viral containing supernatants. Cells were then examined microscopically 48 h later to verify the presence of GFP expression as an indicator of transduction efficiency. Cells were assayed 72 h later to evaluate potential reductions in gene expression by RT-qPCR (compared to non-silencing shRNA).

### Efficiency of secondary sphere formation assay

MKN45, ST2957 and SNU638 CSC enriched sphere-derived cells were maintained in supplemented DMEM/F12 media and after 5-6 days, spheres were harvested and trypsinized to induce sphere dissociation, and single cells were seeded again in ultra-low-attachment 96-well plates for secondary sphere formation (1 cell per well for MKN45, 25 cells for ST2957 and 250 cells for SNU638). Fresh medium was added to the culture every few days, and after 10 days, spheres were counted and Sphere Forming Efficiency (SFE) was calculated as the number of spheres formed relative to the number of seeded cells, and expressed as % mean ± SD. For analysis of the SFE from 1-single cell, MKN45 cells were sorted in ultra-low-attachment 96-well plates using the FACSVantage SE Flow Cytometer (Becton Dickinson). For the experiments with the MAD2 inhibitor, M2i-1, cells were seeded as previously described in the presence of 25 µM M2i-1, which was added again on the fifth day.

### BrdU Incorporation Analysis

MKN45 and ST2957 CSC enriched sphere-derived cells were seeded in ultra-low-attachment 6-well plates (1.5×10^5^ cells/3 mL per well) and allowed to form secondary spheres for 5 days, after which we added 10 µM BrdU for 1 hour. After BrdU incubation, cells were washed with PBS, trypsinized, fixed with 70% cold EtOH and stored at -20°C for at least 2 hours. To stain cells with fluorescence-labeled antibody, cells were washed once with PBS and permeabilized with 3 N HCl for 20 min at room temperature. Then, cells were washed with PBS three times and incubated with blocking solution (1% BSA - 0.05% Tween 20 - PBS) for 15 min at room temperature. Next, we added the FITC mouse anti-BrdU antibody (1:50, 556028 BD Pharmingen) and incubated samples for 1 h at 37°C in the darkness. Later, PI (50 µg/ml) and RNAse (10 µg/ml) were added, without removing the previous solution, and samples were incubated overnight at 4°C in darkness. Cell DNA content and BrdU incorporation were evaluated using a FACSCANTOII Flow Cytometer (Becton Dickinson) and results were analyzed using BD FACSDiVa Software.

### Cell cycle profile analysis

MKN45 2^nd^ generation CSC enriched sphere-derived cells without/with *MAD2L1* downregulation were trypsinized, fixed with 70% cold EtOH and stored at 4°C overnight. Cells were incubated with PI (50 µg/mL) and RNAse (10 µg/mL) for 30 min and DNA content were evaluated using a FACSCANTOII Flow Cytometer (Becton Dickinson). Results were analyzed using BD FACSDiVa Software.

### Cell viability

Viability was determined using a MTS (Promega) staining method. For this, 5×10^3^ adherent cells were seeded per well in 96 multi-well plates and the following day they were treated with various amounts of CDDP (0-10 µg/mL), BLM (0-10 µg/mL) and PXL (0-0.5 µM). CSC enriched sphere-derived cells (1×10^3^ cells for MKN45 and 5×10^3^ cells for SNU638 per well) were seeded in ultra-low-attachment 96-well plates. Four days later, secondary spheres were treated the same way as adherent cells were. 72 h post treatment initiation, 10 μL/well MTS solution was added to each well, and after incubation in the darkness for 1-4 h (37°C, 5% CO_2_), absorbance was recorded at 490 nm.

### Cell migration and invasion assay

Migration and invasion assays were performed in a cell culture insert with a transparent PET Membrane (6.5 mm diameter, 8.0 μm pore size) (transwell migration and invasion assays) (Falcon, Corning Inc., Corning, NY, USA), coated with 6 μg growth factor reduced Matrigel (BD Biosciences, Bedford, MA, USA) for invasion assay. 2^nd^ generation CSC enriched sphere-derived cells (1.5×10^5^) were seeded in the upper chamber, and 20% serum-free conditioned medium from M2-differentiated human macrophages, generated as described in 22, was used as a chemoattractant and placed in the bottom chamber. Cells were allowed to migrate or invade for 48 h at 37°C with 5% CO_2_. Then, non-migrating and non-invading cells were removed using a cotton swab, and the filters were stained with Diff Quik (Dade Behring, Newark, DE, USA). Migratory and invasive cells were counted in 50 fields of maximum migration or invasion under a light microscope at 40× magnification. Representative images were captured using an Axiophot Zeiss microscope.

### Gelatin Zymography

MKN45 CSC enriched sphere-derived cells were seeded in ultra-low-attachment 6-well plates (1.5×10^5^ cells/3 mL in each well) and after five days forming secondary spheres, they were washed with PBS and resuspended in free DMEM/F-12 medium for 48 h. Conditioned medium was harvested and centrifuged at 2000 rpm for 5 min. Then, the conditioned medium was concentrated using centrifugal filter devices (Amicon Ultra-4; Millipore) and stored at -80°C. Media containing 20 µg of total proteins was resuspended in non-reducing sample buffer (62.5 mM Tris-HCl pH 6.8, 1% SDS, 12.5% glycerol, 0.004% bromophenol blue), warmed at 37°C for 15 min and run in a 7.5% polyacrylamide gel containing 0.25% gelatin. After electrophoresis, the gel was washed twice (30 min each) with washing buffer (50 mM Tris-HCl pH 7.5, 2.5% Triton X-100, 5 mM CaCl_2_ and 1 µM ZnCl_2_) with gentle agitation at RT. Next, the gel was washed for 10 min with incubation buffer (50 mM Tris-HCl pH 7.5, 1% Triton X-100, 5 mM CaCl_2_ and 1 µM ZnCl_2_) at RT and then incubated with new incubation buffer at 37°C for 48 h with gentle agitation. After incubation, the gel was stained with staining solution (0.5% Coomassie Brilliant Blue R-250 (161-0400 Bio-Rad Laboratories), 40% methanol, 10% acetic acid) for 30 min - 1 h and destained with destaining solution (40% methanol, 10% acetic acid) until gelatinolytic bands appeared as a clear white zone against a blue background. The intensity of the gelatinolytic bands was quantified using ImageJ software.

### Tumor xenograft model

MKN45 cells were trypsinized and 5×10^3^ cells were resuspended in 50 µL medium with 40% Matrigel™ (Corning), and then subcutaneously injected into the flanks of female 5-week-old nude mice (Janvier Labs). To analyze the effect of the MAD2 inhibitor M2i-1, we added 25 µM to the cell preparation before injecting into mice. Tumor growth and volumes were monitored every few days for up to 4 weeks with a caliper. Tumor volume (mm^3^) was computed using the formula: 0.5 × D1^2^ × D2, where D1 and D2 are the width or the largest diameter and the length or the smallest diameter of a given tumor, respectively. After sacrifice by cervical dislocation, tumor mass was resected and trimmed; one fragment was embedded in OCT and frozen, and the others were directly frozen for protein and mRNA extraction and cDNA synthesis (as previously described, with an initial step of homogenization). OCT-embedded tumors were cut through the maximum diameter section plane and 5 µm-thick sections were obtained using a Leica Cryostat 1950 for H&E staining. Pictures of each slide were taken with a Nikon Eclipse 90i microscope and ImageJ software was used to stitch all microscopic fields together so as to make up the complete section. The viable tumor surface was calculated by drawing the viable areas using ImageJ software, which are distinguishable from necrotic areas. All animal experiments were conducted in accordance with FELASA guidelines and approved protocols. Mice were housed according to institutional guidelines and all experimental procedures were performed in compliance with the institutional guidelines for the welfare of experimental animals approved by the Universidad Autónoma de Madrid Ethics Committee (CEI 60-1057-A068) and La Comunidad de Madrid (PROEX 335/14) and in accordance with the guidelines for Ethical Conduct in the Care and Use of Animals as stated in The International Guiding Principles for Biomedical Research involving Animals, developed by the Council for International Organizations of Medical Sciences (CIOMS).

### Statistical analysis

All data are presented as mean ± standard deviation (SD) after performing three independent experiments, unless otherwise stated. Statistical significance (at *P* < 0.05) was determined using one-way ANOVA, post hoc comparisons, Bonferroni's test, or two-tailed Student's t-test, using GraphPad Prism version 5.0c (San Diego, California, USA).

## Results

### Isolation and characterization of gastric cancer *stem* cells (GCSCs)

**Figure 1:**
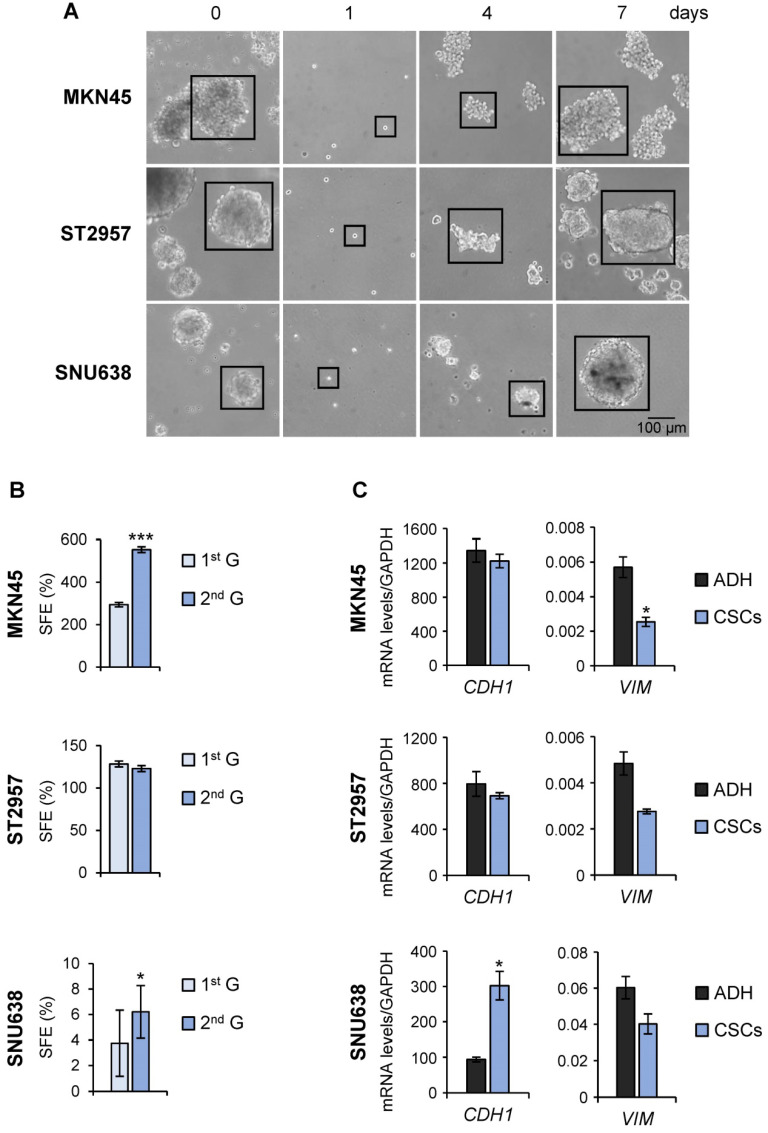
** Isolation of CSCs from GC cell lines. (A)** Sphere formation assay for MKN45, ST2957 and SNU638 GC cell lines. Cells were grown in ultra-low-attachment 6-well plates with DMEM/F-12 medium (3000 cells per well) containing specific supplements. Images are representative of 1^st^ generation spheres taken at day 7 (time 0) and 2^nd^ generation spheres taken 1, 4 and 7 days after plating. Scale bar: 100 µm. **(B)** Sphere formation efficiency in GCSCs. 1^st^ and 2^nd^ generation CSC enriched sphere-derived cells were seeded as follow: 10 cells/well for MKN45 and ST2957 and 100 cells/well for SNU638, and spheres were counted 10 days later. Graphs show the percentage of the total number of spheres formed/total number of cells seeded (SFE: sphere formation efficiency). **(C)** RT-qPCR was performed to measure E-cadherin (*CDH1*) and Vimentin (*VIM*) mRNA relative levels on samples from the three GC cell lines and their CSCs at day 7. Each gene was normalized to *GAPDH* and data were calculated as relative to human fibroblasts (set as 1.0). Three independent experiments were performed with similar results in (B) and (C). Statistical significance was evaluated by a one-way ANOVA, followed by post hoc comparison (Bonferroni´s test, *P < 0.05; **P < 0.01). ADH: adherent cells. CSCs: enriched CSC cultures.

To evaluate the utility of GC cell lines as suitable models for CSC-based studies, GC cell lines (AGS, MKN45, SNU1, SNU638 and ST2957) were first plated in serum‐free medium under low‐adherent conditions to assess their capacity to form floating three‐dimensional spheroids, enriched in cancer *stem*-like cells (CSCs), hereafter referred to as tumorspheres or gastrospheres. MKN45, ST2957 and SNU638 formed tumor spheres but not AGS or SNU1 cell lines (Figure 1A and Figure S1A). To confirm the self‐renewing ability of spheroid forming cells, we enzymatically dissociated the primary spheres into single cells and re-cultured them to generate sub‐spheroid bodies again, and passaged them twice for at least 3 weeks in serum‐free CSC-specialized media under non-adherent conditions. Our results showed that spheroid body‐forming cells retain the ability to self‐renew (Figure 1A). Interestingly, the sphere body-forming efficiency increased in MKN45 and SNU638 cell lines in 2^nd^ generation, but the maximum CSC enrichment was achieved in ST2957 in the first generation (Figure 1A). Moreover, to study the differentiation of GCSCs we disaggregated the spheres and cultivated individual cells in serum‐containing medium under adherent conditions. Five days after seeding, we observed obvious phenotypical changes in our cells, which now showed an adherent morphology (Figure S1B). Finally, we evaluated the mesenchymal state of these CSC-enriched spheres by measuring the expression of 2 EMT-associated genes. This analysis revealed an increase of the expression of E-cadherin (*CDH1*), the major signature gene for epithelial cells, in SNU638^CSCs^, and a reduction in the expression of the mesenchymal marker vimentin (*VIM*) in all cells, reaching significance for MKN45^CSCs^ (Figure 1C).

**Figure 2:**
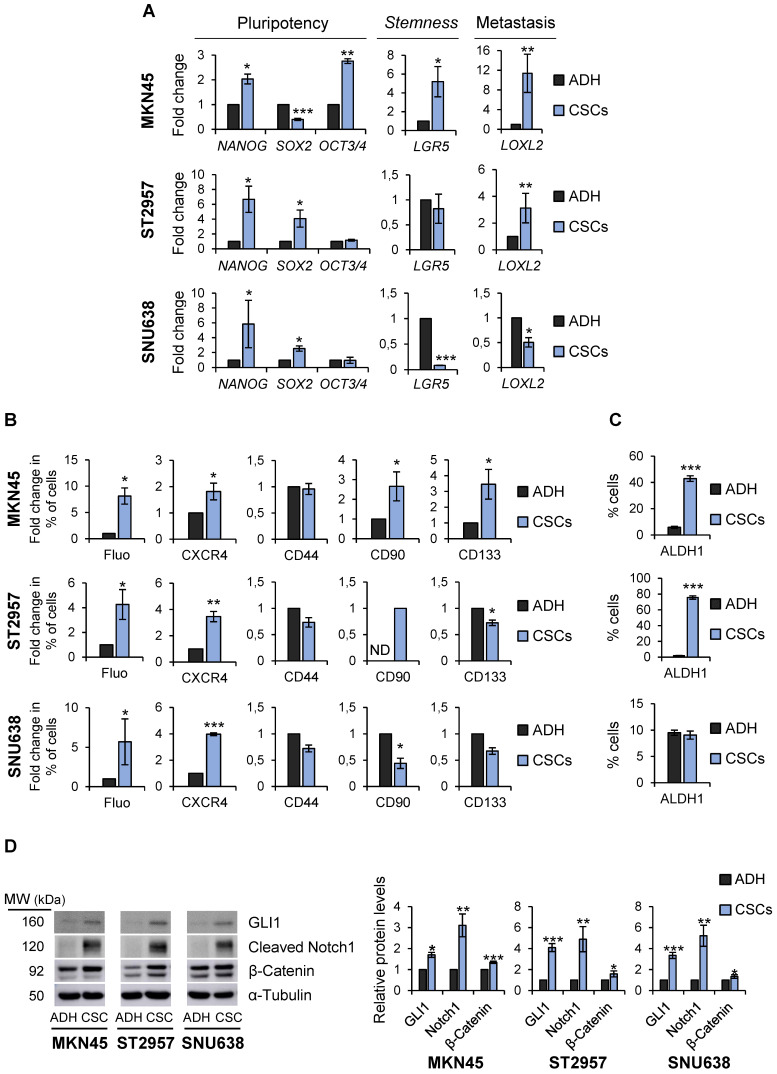
** Characterization of GCSCs. (A)** Relative levels of mRNA transcripts for pluripotency and *stemness* markers. The relative expression levels of *NANOG*, *SOX2*, *OCT3*/4, *LGR5* and *LOXL2* mRNAs were quantified by RT-qPCR in MKN45, ST2957 and SNU638 cell lines cultured in adherence (ADH) or as spheres (CSCs). Each gene was normalized to *GAPDH*. Fold changes were calculated and compared to ADH (set as 1.0). At least three independent experiments were performed with similar results.** (B)** Flow cytometric analysis for CXCR4, CD44, CD90 and CD133 cell surface expression and autofluorescence (Fluo) in the 3 GC cell lines cultured in adherence (ADH) or as spheres (CSCs). The histograms summarize the percentage of CXCR4-, CD44-, CD90-, CD133- and autofluorescent-positive cells from three different experiments. Fold changes were calculated compared to ADH (set as 1.0). **(C)** ALDH activity profile. MKN45, ST2957 and SNU638 cell lines cultured in adherence (ADH) or as spheres (CSCs) and were counter-stained with AldeRed to determine the frequency of ALDH-positive cells by flow cytometry, in the presence or absence of the ALDH inhibitor (DEAB). ALDH activity is expressed as mean of the percentage of positive cells of 3 experiments. **(D)** Activation of signaling pathways in CSCs. Representative Western Blot analysis for GLI1, cleaved Notch1 and β-catenin in the 3 GC cell lines cultured in adherent and sphere conditions. Graphs show the fold change of protein levels compared to ADH expression obtained in three different experiments performed under the same conditions. Protein levels were normalized with α-tubulin. The statistical significance was evaluated with two-tailed Student's t-test (*P < 0.05; **P < 0.01; ***P < 0.001).

To more definitively determine the *stem*-like state of GCSCs, the expression of pluripotency and *stemness* markers in sphere-forming cells compared with adherent cells was analyzed. RT-qPCR analysis showed that *NANOG* was sharply up-regulated in all GCSC-enriched cultures studied by 2, 6.6 and 5.8 fold-times in MKN45, ST2957 and SNU638, respectively*.* Furthermore,* SOX2* mRNA levels increased in ST2957^CSCs^ (4 fold-times) and SNU638^CSCs^ (2.5 fold-times), while MKN45^CSCs^ showed an increase in the expression of *OCT3/4* (2.7 fold-times) (Figure 2A). To verify our RT-qPCR results, we checked SOX2 and NANOG protein levels in these cell lines, and our WB results corroborated the above mRNA data (Figure S2A). We also checked other CSC markers, such as *LGR5*, whose expression increased around 5 times in MKN45^CSCs^. In addition, a clear and significant rise in the expression of the metastatic EMT-associated gene *LOXL2* both in MKN45^CSCs^ (11.3 fold-times) and ST2957^CSCs^ (3 fold-times) cell lines was observed (Figure 2A).

Finally, the expression of CSC markers was assessed by flow cytometry. First, we explored autofluorescence, a recently described CSC marker 21, which is the result of riboflavin accumulation in ABCG2-coated intracellular vesicles. Compared to their adherent cell counterparts, we observed an increase in MKN45^CSCs^, ST2957^CSCs^ and SNU638^SCs^ autofluorescence by 8, 4.25 and 5.7, respectively. Moreover, we investigated the expression of the plasma membrane marker CXCR4 and observed an increase in the percentage of positive cells in MKN45, ST2957 and SNU638, by 1.8, 3.4 and 3.9 fold-times, respectively. CD90 was only expressed in ST2957^CSCs^ and not in adherent cells and increased by 2.65 fold-times in MKN45^CSCs^. CD133 was 3.45 times higher in MKN45^CSCs^ and we did not appreciate any modification in the expression of CD44 (Figure 2B and Figure S2B). We also analyzed ALDH1 activity, a functional CSC marker, and observed that CSCs from MKN45 and ST2957 showed a significant and pronounced increase in ALDH1 activity (Figure 2C and Figure S2C). Finally, we analyzed three well known signaling pathways commonly activated in CSCs: Hedgehog, Notch and Wnt/β-catenin. WB analysis of one representative component of each pathway (GLI1, cleavage-NOTCH1 and β-CATENIN, respectively) showed that all of these pathways are activated in CSCs enriched from the three GC cell lines (Figure 2D).

Altogether, our data suggests that these GC cell lines contain a pool of heterogeneous cells which show characteristics of CSCs, although differences between cells lines exist, as would be expected. Nevertheless, the GCSCs from MKN45, ST2957 and SNU638 have the ability of self-renewal and express, as common CSC markers, *NANOG*, CXCR4 and autofluorescence.

### Mitotic checkpoint

**Figure 3:**
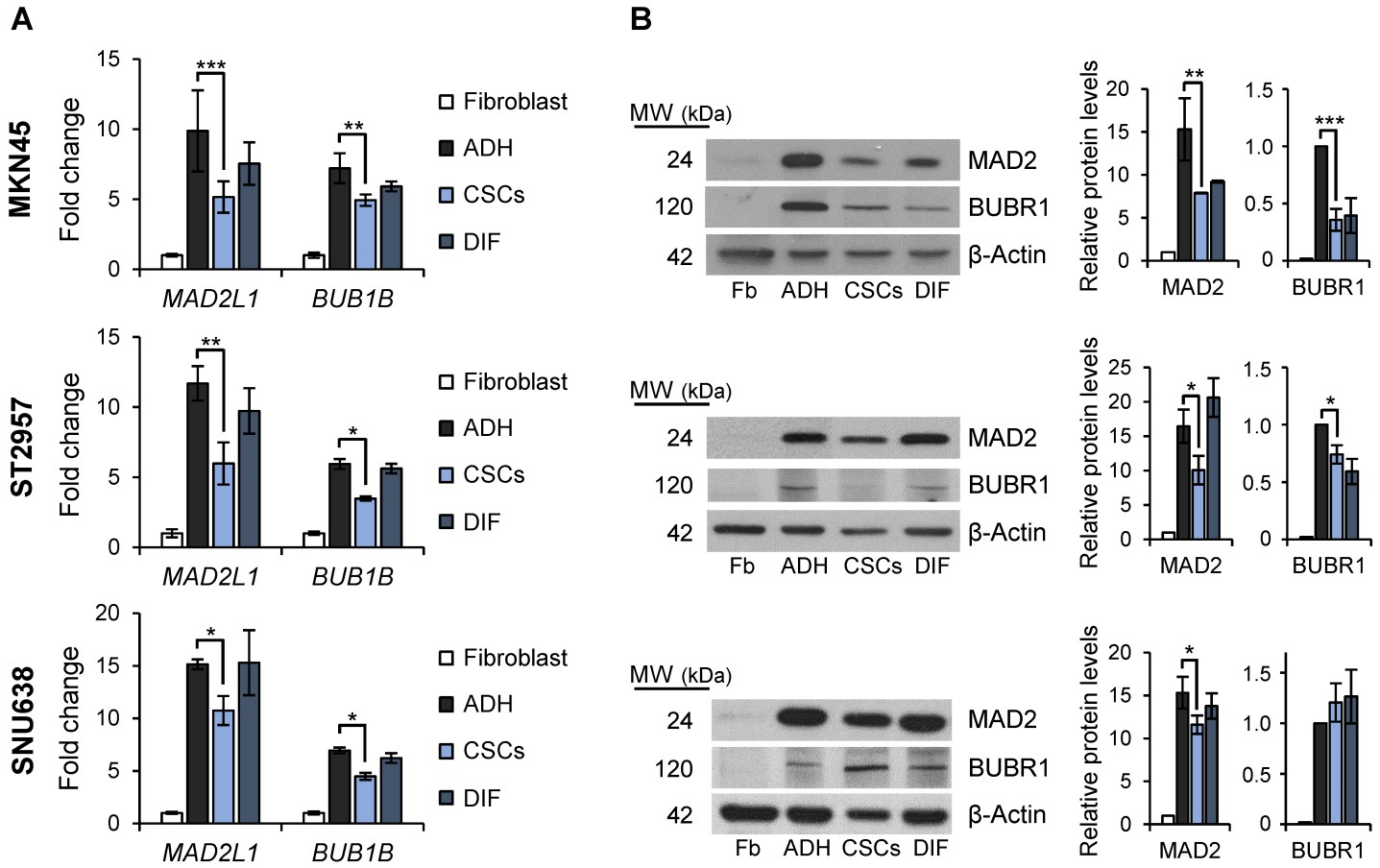
** SAC key protein MAD2 and BUBR1 levels in GCSCs.** RT-qPCR **(A)** and WB **(B)** analysis for *MAD2L1* (MAD2) and *BUB1B* (BUBR1) in the 3 GC cell lines cultured in adherence (ADH), as sphere (CSCs) or re-differentiated cells (DIF). Each gene was normalized to *GAPDH*. Protein levels were normalized with β-actin. Data are shown as expression relative to human fibroblasts (Fb). The experiments were repeated three times with similar results. Statistical significance was evaluated with one-way ANOVA, followed by post hoc comparisons (Bonferroni´s test, *P < 0.05; **P < 0.01; ***P < 0.001).

Mitosis plays important roles in balancing the *stem* cell and non-*stem* cell populations via regulation of symmetric division (i.e. self-renewal) and asymmetric division (i.e. differentiation, giving rise to progenitor cells). We asked if the mitotic checkpoint proteins MAD2 and BUBR1 also play a role in the tumorigenic process of GCSCs. To this end, we quantified *MAD2L1* and *BUB1B* mRNA and protein levels in the GC cell lines cultured both in adherent conditions or as spheres, and also evaluated and compared levels to primary human non-tumoral fibroblasts as non-tumoral gastric cells are unavailable. Our results showed that while *MAD2L1* and *BUB1B* mRNA levels are upregulated in both adherent cells and 2^nd^ generation GCSC-enriched spheres compared to fibroblasts, adherent differentiated cultures have higher expression of both transcripts versus GCSC-enriched spheres: 9.8 vs 5.2 fold-times in MKN45, 11.6 vs 5.8 in ST2957 and 15.1 vs 10.7 in SNU638 for *MAD2L1*, and 7.2 vs 4.9 fold-times in MKN45, 5.9 vs 3.5 in ST2957 and 6.9 vs 4.5 in SNU638 for *BUB1B* (Figure 3A). In order to test if this overexpression was acquired during the differentiation process, we re-seeded 2^nd^ generation GCSCs under adherent conditions (DIF) (as described for Figure 1B) and quantified the mRNA levels of these key mitotic proteins. Our results showed that re-differentiated cells upregulated *MAD2L1* and *BUB1B* almost to the same levels as adherent cells (i.e. 7.6, 9.7 and 15 fold-times of *MAD2L1* mRNA levels in MKN45, ST2957 and SNU638, respectively). We verified these data by WB analysis, and confirmed the same trend for MAD2 and BUBR1 (Figure 3B). The sum of these data suggests that GCSCs overexpress MAD2 and BUBR1, which further increases during differentiation (i.e. asymmetric division).

### *MAD2L1* interference modulates *stemness* characteristics

**Figure 4:**
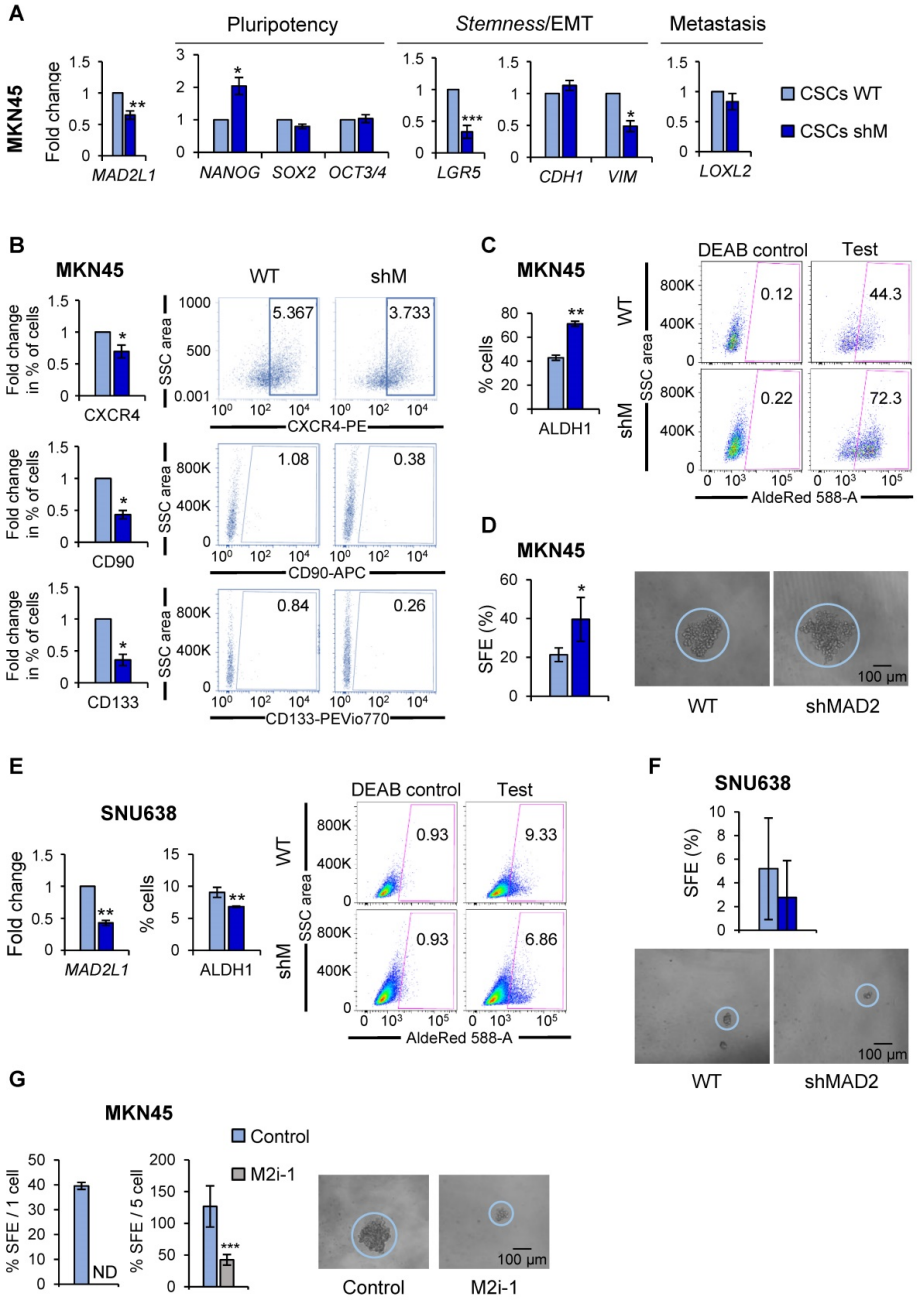
**MAD2 modulates pluripotency and *stemness* ability in GC cell lines. (A)** The interference of *MAD2L1* in MKN45^CSCs^ (shM) was confirmed by RT-qPCR. The relative expression levels of *NANOG*, *SOX2*, *OCT3*/4, *LGR5*, *CDH1*, *VIM* and *LOXL2* mRNAs were quantified by RT-qPCR in the MKN45^CSCs^. Each gene was normalized to *GAPDH*. Fold changes were calculated relative to the non-interfered CSCs (WT). At least, three independent experiments were performed with similar results.** (B)** Flow cytometric analysis of CXCR4, CD90 and CD133 cell surface markers in MKN45^CSCs^ after interference of *MAD2L1*. The histograms summarize the percentage of CXCR4-, CD90- and CD133-positive cells in sphere cultures from three different experiments. Fold changes were calculated compared to WT. Representative plots with the percent-positive cells present within the single-cell, live and debris-free population are shown. **(C)** ALDH activity profile. MKN45^CSCs^ after interference of *MAD2L1* were counter-stained with AldeRed to determine the frequency of ALDH-positive cells by flow cytometry, in the presence or absence of the ALDH inhibitor (DEAB). ALDH activity is expressed as mean of the percentage of positive cells of 3 experiments. Representative plots are shown, in the presence or absence of the ALDH inhibitor (DEAB), with the percent-positive cells present within the single-cell, live and debris-free population. **(D)** Secondary sphere formation efficiency in MKN45^CSCs^ with reduced MAD2 levels. 1 cell/well was seeded by sorting and spheres were counted 10 days later. Graphs show the percentage of the total number of spheres formed/total number of cells seeded (SFE: sphere formation efficiency). Right panels show representative images of tumorspheres. Scale bar: 100 µm. **(E)** RT-qPCR for *MAD2L1* and ALDH activity profile in SNU638^CSCs^. mRNA levels of *MAD2L1* and frequency of ALDH-positive cells were quantified as in (A) and (C), respectively. **(F)** Secondary sphere formation assay in SNU638^CSCs^ with reduced MAD2 levels. 250 cells/well were seeded and SFE was calculated as in (D). Panels below show representative pictures of tumorspheres. Scale bar: 100 µm. **(G)** Secondary sphere-forming capacity of MKN45^CSCs^ in presence of the MAD2 inhibitor, M2i-1 (25 µM), after ten days, 1 cell/well in left panel (by sorting) and 5 cells/well in right panel. Graphs show the percentage of the total number of spheres formed/total number of cells seeded. Right panels show representative images of tumorspheres when seeding 5 cells/well. Scale bar: 100 µm. The experiments (D, E, F, G), were repeated three times with similar results. Statistical significance was evaluated with two-tailed Student's t-test (*P < 0.05; **P < 0.01; ***P < 0.001).

These results prompted us to study the role of MAD2 in gastric tumorigenesis taking into account the fundamental importance of this protein in modulating mitotic checkpoint and asymmetric division 23. Towards this end, we chose to continue with MKN45^CSCs^ as this poorly differentiated adenocarcinoma cell line showed the most consistent results at the level of CSC phenotypes (Figure 2). We used an shRNA targeting *MAD2L1* (shM), and performed RT-qPCR analysis to study the effect of MAD2 downregulation on the expression of pluripotency and *stemness* markers in MKN45^CSCs^, as described above. First, we confirmed the efficiency of *MAD2L1* interference in MKN45 CSC 2^nd^ generation-enriched cultures (Figure 4A). Next, we analyzed the cell cycle of shM versus non-interference (WT) to determine the effect of MAD2 silencing on GCSC cell cycle progression. We observed that the distributions of cells in different phases of the cell cycle were not significantly different comparing WT vs shM MKN45^CSCs^ (Figure S3A). Moreover, we analyzed BrdU incorporation, and observed a slightly higher incorporation of BrdU index after MAD2 downregulation (1.5 fold-times) (Figure S3B). To further analyze the cell cycle, we studied proteins directly involved in cell cycle control. Our results showed no differences in cyclin levels (Cyclin D1, Cyclin A2, Cyclin B1) or Cdk-kinases (CDK1 and pCDK1^Y15^), and a slight increase in HistoneH3^Ser10^ phosphorylation (Figure S3C). Thus, we concluded that MAD2 silencing has a marginal effect on MKN45^CSCs^ cell cycle progression.

At the level of gene expression; however, we observed significant but variable changes in marker expression after *MAD2L1* downregulation. Except for *NANOG,* pluripotency-associated genes were unaffected in shM versus WT MKN45^CSCs^. *LGR5*, also significantly decreased in shM versus WT. At the level of EMT- and metastasis-associated genes, no changes were found in *CDH1* or *LOXL2* expression; however, a significant reduction in *VIM* levels were observed after *MAD2L1* interference (Figure 4A). At the level of the percentage of cells expressing CSC surface markers, we observed a significant reduction in the percentage of CXCR4, CD90 and CD133-positive cells. Autofluorescence was not assessed as shM cells express GFP, making it impossible to assess autofluorescence (Figure 4B).

Since the aforementioned data indicated a potential decrease in the CSC population, we next assessed CSC functional properties (ALDH1 activity and self-renewal). To our surprise, ALDH1 activity remained high in MKN45, showing almost 2-times more activity in shM versus WT MKN45^CSCs^ (Figure 4C). Likewise, SFE for WT was approximately 21.35% while for shM a SFE of around 39.58% was calculated (Figure 4D).

It is important to note that although *MAD2L1* silencing in MKN45 cells was significantly reduced, silencing was not > 50%. Additional silencing attempts to further reduce MAD2 levels were unsuccessful (data not shown), which we attribute to lethal effect in MKN45 cells, as these cells expressed the lowest levels of MAD2 across the three GC cells lines initially tested. Therefore, *MAD2L1* was silenced in SNU638 cells, which expressed the highest levels of MAD2 (Figure 3A), and we achieved a greater than 50% reduction in *MAD2L1* (Figure 4E). Interestingly, with a more robust reduction in MAD2, we observed in shM SNU638^CSCs^ a significant inhibition in ALDH1 activity (Figure 4E) and a reduction in SFE (Figure 4F), indicating that the levels of MAD2 are key to impact GCSC properties. To test this hypothesis in MKN45^CSCs^, we decided to abolish the activity of MAD2 by using the inhibitor M2i-1 in MKN45^CSCs^ in lieu of silencing *MAD2L1*. Following M2i-1 treatment, we analyzed sphere formation capacity via a limiting dilution assay and index sorting. Our results showed that the activity of MAD2 is essential for CSC phenotypes, and only when MAD2 levels are reduced to suboptimal levels or its activity is completely inhibited do GCSC lose their inherent *stem*-associated phenotypes (Figure 4G).

**Figure 5:**
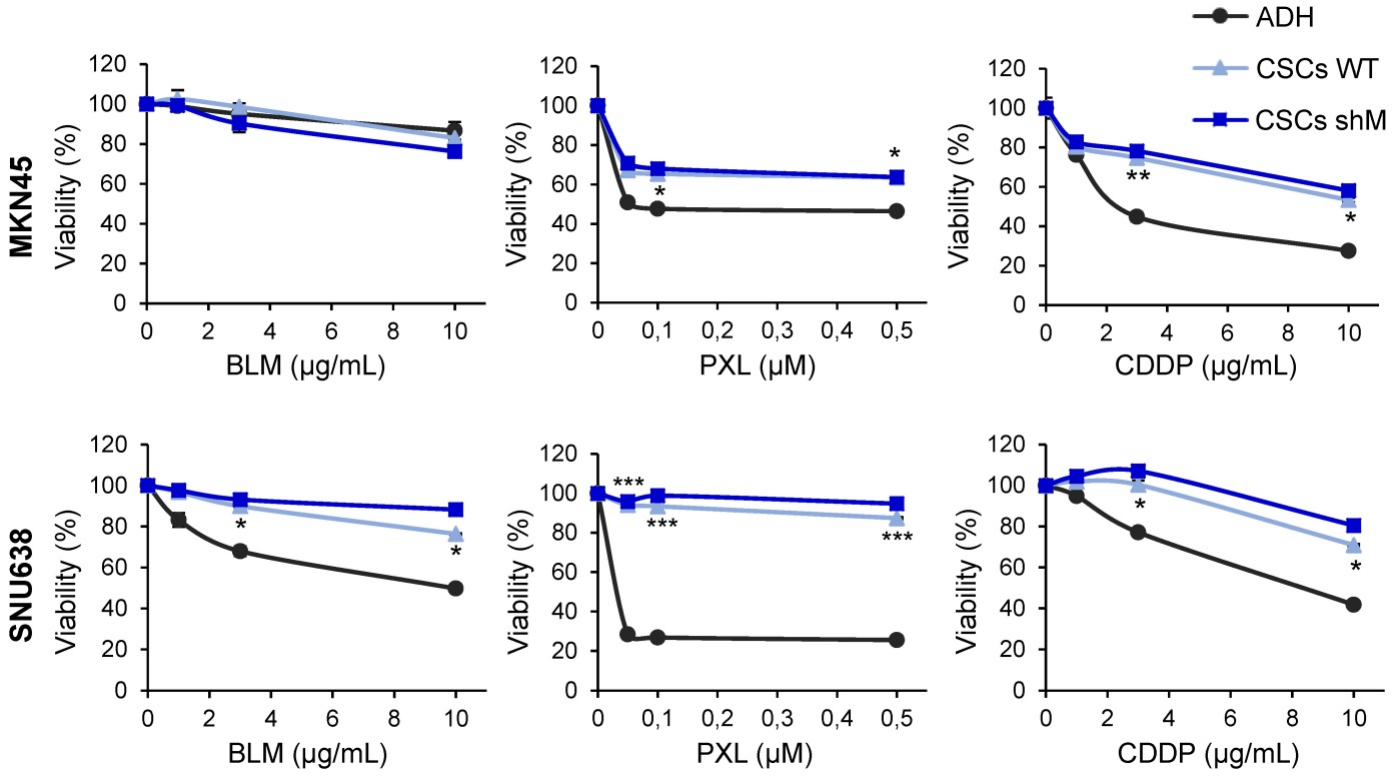
** Chemoresistance in GCSCs.** Cell viability was quantified using MTS assay after 72 h of bleomycin (BLM), paclitaxel (PXL) and cisplatin (CDDP) treatment in increasing concentrations in MKN45 and SNU638 cell lines cultured in adherence (ADH) or as sphere (CSCs), after MAD2 downregulation (CSCs shM) or not (CSCs WT). Results are presented as the percentage of viable cells relative to untreated cells. Data represent the mean values obtained in three experiments performed in quadruplicate. The statistical significance was evaluated with one-way ANOVA, followed by post hoc comparisons (Bonferroni´s test, *P < 0.05; **P < 0.01; ***P < 0.001).

Next, as chemoresistance is another hallmark trait of CSCs, we asked whether sensitivity to standard chemotherapeutics was affected as a consequence of modulating MAD2 levels in MKN45^CSCs^ and SNU638^CSCs^. We tested cell survival using an MTS assay, 72 h after the treatment with increasing amounts of bleomycin (0-10 µg/mL), paclitaxel (0-0.5 µM) and cisplatin (0-10 µg/mL). As expected, GCSCs were more resistant to the indicated treatment compared to adherent cells. Interestingly, reduced levels of MAD2 did not have any significant effect on survival regardless of the level of *MAD2L1* silencing (Figure 5).

Altogether, the above results suggest that MAD2 levels have an impact on the *stemness* phenotype in GCSCs, but not in the therapy response, and a certain threshold of MAD2 levels and/or activity is required for the GCSC state.

### MAD2 modulates migration, invasion and *in vivo* tumorigenesis

**Figure 6:**
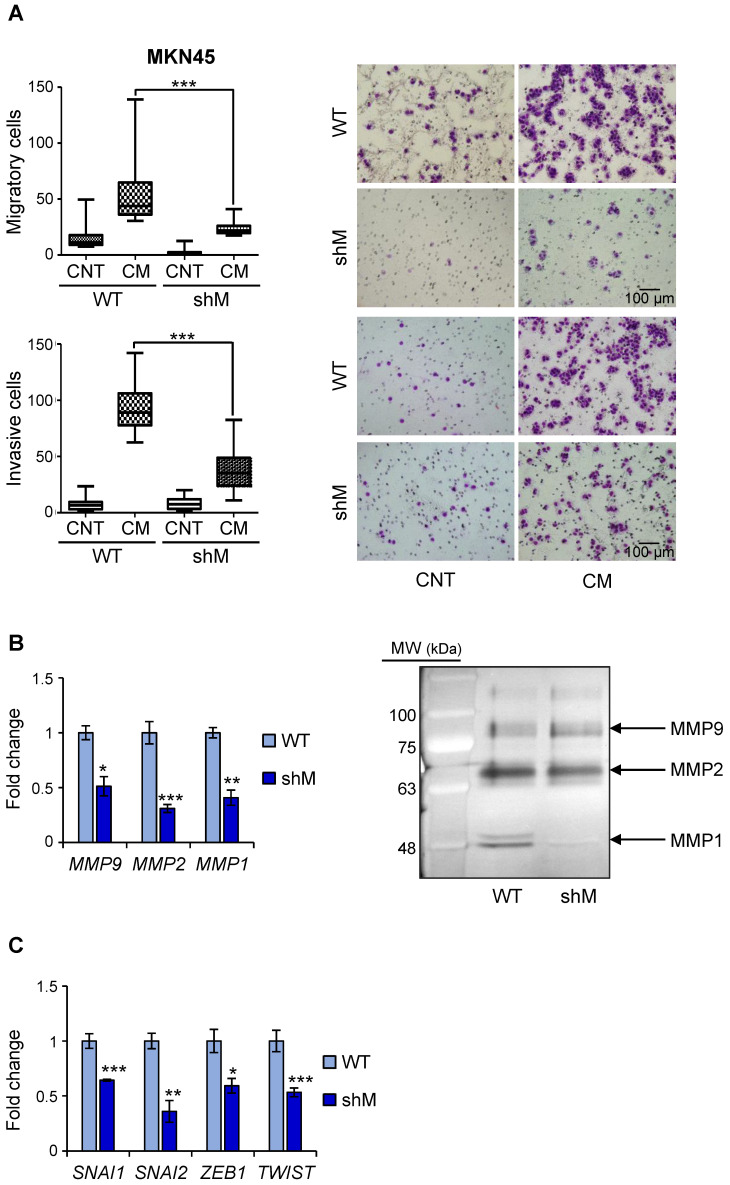
** MAD2 levels regulate migration and invasion in MKN45^CSCs^. (A)** Transwell migration (top) and invasion (bottom) assay in MKN45^CSCs^ after MAD2 downregulation. Graphs show the quantification of stained migratory and invasive cells, respectively, from CSCs WT and shM using the transwell assay, without and with 20% serum-free conditioned medium from M2-differentiated human macrophages (CM) at 48 h. Representative photographs of the experiment are shown on the right. Scale bar: 100 µm. **(B)** Analysis of MMPs in MKN45^CSCs^ with reduced MAD2 levels. The relative expression levels of *MMP9*, *MMP2* and *MMP1* mRNAs were quantified by RT-qPCR. Each gene was normalized with *GAPDH*. Fold changes were calculated compared to WT. On the right, gelatin zymography assay of MMP9, MMP2 and MMP1 activities. **(C)** RT-qPCR analysis for EMT genes *SNAI1*, *SNAI2*, *ZEB1* and *TWIST* as in (B). At least, three independent experiments were performed with similar results. The statistical significance was evaluated with one-way ANOVA, post hoc comparisons, Bonferroni´s test, or two-tailed Student's t-test (*P < 0.05; **P < 0.01; ***P < 0.001).

Having observed a reduction in *VIM* in shM MKN45^CSCs^, we performed *in vitro* assays to analyze migration and invasion under conditions of *MAD2L1* downregulation. Our results showed that depleting MAD2 reduces both the median migration and invasion in a transwell assay (43.75 to 26 cells/field and 89 vs 23.5 cells/field, respectively) (Figure 6A). Identical results were also observed for shM SNU638^CSCs^, indicating that maximum MAD2 silencing is not necessary to affect migration and invasion (Figure S4).

Since matrix MMPs are crucial for the progression of the invasion process, we analyzed the expression in MKN45^CSCs^ of some of the more common MMPs implicated in invasion ability: MMP9, MMP2 and MMP1. Our results showed that in shM MKN45^CSCs^ the mRNA levels for those MMPs dropped to less than half the values of those measured in WT MKN45^CSCs^ (Figure 6B). We also studied the activity of MMPs in conditional medium from MKN45^CSCs^ by zymography assay, and observed that MMP1 and MMP2 activity were reduced under MAD2 downregulation (Figure 6B). Finally, analyzing mRNA levels for known EMT genes we corroborated that key transcription factors involved in the EMT process (i.e. *SNAI1*, *SNAI2*, *ZEB1* and *TWIST*) are downregulated after *MAD2L1* interference (Figure 6C).

**Figure 7:**
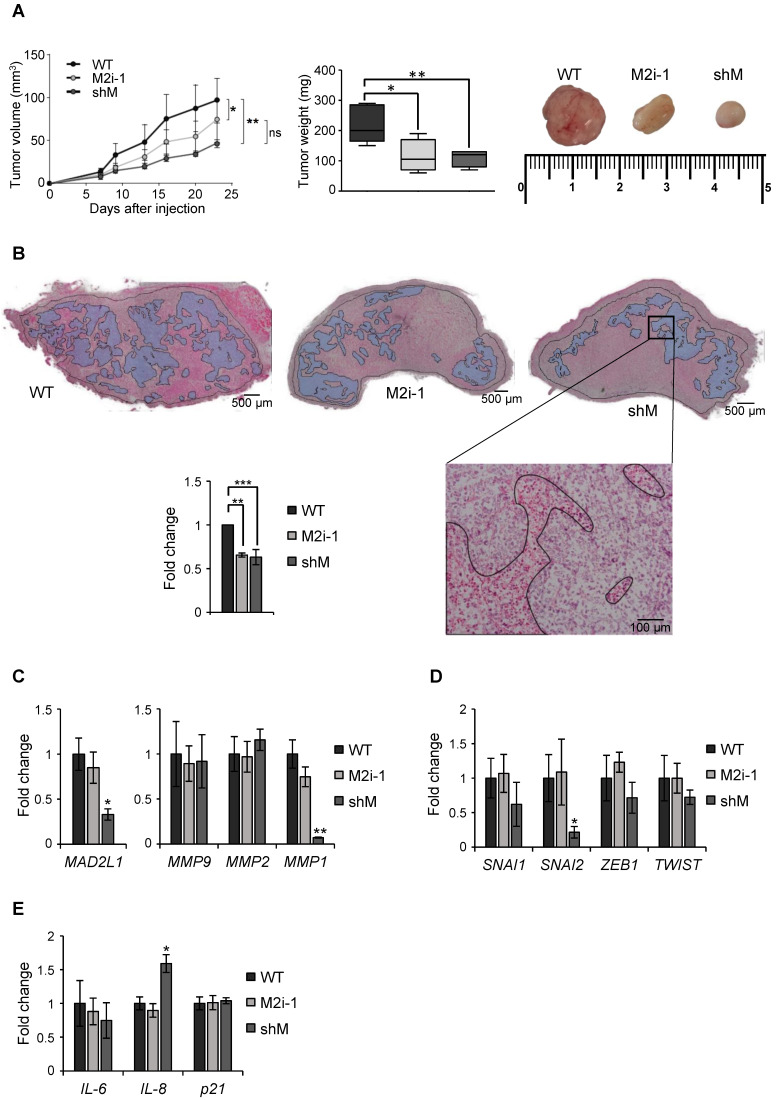
** MAD2 downregulation reduces tumorigenesis *in vivo* in MKN45 cell line. (A)** MKN45 cells (WT), after *MAD2L1* downregulation (shM) and in presence of the MAD2 inhibitor M2i-1 (M2i-1), were subcutaneously injected (5 × 10^3^ cells), into the flanks of nude mice. Growth of tumors was monitored by measurement of tumor size at specific time points. Tumors were dissected and weighed at the end of the experiment and results are represented in the middle panel. Representative images from the tumors are shown in the upper right panel. **(B)** Hematoxylin-eosin staining in tumor sections from MKN45 WT, M2i-1 and shM tumors with viable areas traced (Magnification 2x, Scale bar: 500 µm). Detail of tumor tissue with necrotic areas are traced (Magnification 10x. Scale bar: 100 µm). On the left, graph showing the viability of the tumors relative to wild-type cells-tumors (WT). Data represent the mean values obtained from three sections per tumor cut by the maximum diameter to be representative. **(C-E)** RT-qPCR analysis for *MAD2L1* and *MMP9*, *MMP2* and *MMP1* **(C)**, EMT genes *SNAI1*, *SNAI2*, *ZEB1* and *TWIST* **(D)** and senescence-related genes *IL-6*, *IL-8* and *p21*** (E)** in tumors resected for the experiment in (A). Each gene was normalized to *GAPDH*. Fold changes were calculated compared to WT. Data represent the mean values obtained from three different tumors for each condition. Error bars represent ± SEM. The statistical significance was evaluated with one-way ANOVA, followed by post hoc comparisons (Bonferroni´s test, *P < 0.05; **P < 0.01; ***P < 0.001).

Finally, a xenograft assay was performed in immunocompromised mice to evaluate the role of MAD2 in tumor take and progression. MKN45 WT and MKN45 shM cells were subcutaneously injected in both flanks of immunocompromised mice (n=8). Tumor incidence was unchanged regardless of MAD2 levels (8/8), however the tumors continuously increased in size in the WT; while in shM tumor growth was significantly delayed. We also tested the potential effect of M2i-1 on tumor growth, by pre-treating WT cells prior to injection. We observed that tumor volume was intermediate between WT and shM cells, probably due to a reversible effect of the inhibitor. Tumor weight correlated with tumor volume, with an average of 220 mg for WT cells vs ~100 mg for those tumors where activity or expression of MAD2 was inhibited (Figure 7A). H&E staining showed large necrotic areas in those tumors with MAD2 activity modulated compared with WT, and quantification of the viable tumor surface indicated a reduction of approximately 40% when MAD2 was targeted (Figure 7B).

Upon termination of the experiment, we performed *ex vivo* analyses of the resected tumor to assess specific parameters. First, we verified that sh-mediated interference was maintained along the course of the experiment by RT-qPCR analysis of *MAD2L1*. We then analyzed the levels of MMPs between tumors and observed that MMP1 was reduced in the shM group, while there was no significant modification in MMP2 or MMP9 (Figure 7C). Analysis of *SNAI1*, *SNAI2*, *ZEB1* and *TWIST* showed a decreasing trend in shM, with only *SNAI2* reaching significance (Figure 7D). Previous works showed that one of the consequences of MAD2 reduction is induction of senescence 13. Therefore, we tested molecular markers for senescence (IL6, IL8 and p21), and we only found a moderate increase in IL8 expression (Figure 7E). In addition, p53 activation was not observed under the conditions of our study (data not shown). Altogether, our results indicate that MAD2 levels control migration/invasion and tumor growth *in vitro* and *in vivo*, respectively, through the reduction of MMPs and transcription factors involved in EMT and *stemness*. Furthermore, this delay in growth appears not to be related with senescence.

## Discussion

GC is the third leading cause of worldwide cancer deaths, trailing lung and colorectal cancer in overall mortality. While GC survival rates have steadily improved over the past 40 years thanks to earlier detection and better treatment options, high incidence and mortality contributes to a still dismal prognosis 1. Previous reports have shown that CSCs are responsible for cancer recurrence and resistance to therapy 6. Although a significant correlation between Chromosome instability (CIN), CSCs and GC has attracted much attention from researchers, the detailed mechanism by which CIN mediates its effects on GC progression remains largely unknown. In the present study, we demonstrated for the first time that MAD2 levels, a key mitotic checkpoint protein, alters the phenotype and behavior of gastrospheres, specifically *stemness* characteristics, EMT, MMPs and tumorigenic capacity.

MAD2 overexpression promotes aneuploidy, tumorigenesis and leads to lung tumour relapse in mice 24, 25. In human cancers, high expression of MAD2 is also a common feature, with conflicting data regarding prognosis 13. These contradictory data come from the fact that the tolerance of CIN is tissue specific; thus, the absence of MAD2 is incompatible with embryonic development 26 and toxic to hair follicle *stem* cells 27, but tolerated by basal epidermal cells 27, T cells and hepatocytes 28. We propose here that there is a threshold of MAD2 levels in the tumor and in GCSCs, and levels that fall below the “minimum” will affect GCSCs *stemness* characteristics, minimizing their self-renewal and tumorigenic potential. GCSCs express MAD2, although to levels less than that observed in their non-CSC adherent counterparts. Thus, CSCs need "a certain level MAD2 protein" for specific *stem*-related functions (i.e. self-renewal). We hypothesize that this threshold varies across cell lines. For example, MKN45 cells are less tolerable to low, prolonged and potent genetic silencing of MAD2. When levels are too low, cells die and only those cells with levels just above the critical threshold survive. These surviving cells show modulation of several CSC-related properties (e.g. *stem*-associated gene and cell surface maker expression, migration, invasion and tumor growth) but others are only marginally inhibited (e.g. self-renewal). Other cells, such as SNU638, better tolerate MAD2 inhibition. SNU638 cells showed more efficient MAD2 interference compared to MKN45 cells. In these cells, SFE formation was significantly inhibited. Finally, it is important to note that using a MAD2 pharmacological inhibitor in lieu of shRNA-mediated MAD2 silencing; we show that MAD2 activity is required for SFE formation in MKN45 cells. In addition, we hypothesize that MAD2 levels influence the balance between symmetric and asymmetric division during mitosis 29. For instance, in the hematopoietic system, a decrease in MAD2 levels favour asymmetric rather than symmetric cell division and therefore an increase in the number of progenitors that subsequently will differentiate into the corresponding cell types 10. Moreover, MAD2 mutants in flies with abnormal number of centrosomes shows defects in spindle position and tumor reduction associated with asymmetric division and differentiation due to a decrease in the number of proliferative adult intestinal *stem* cells (ISC), which leads to premature differentiation 30. MKN45 CSCs show multipolar mitosis, thus, we can hypothesize that addition of aneuploidy to a tumour-permissive background would influence the tumorigenic capacity.

Our data highlights that decreasing MAD2 in GC may favour a heterogenic CSC population, with a mixed phenotype of more differentiated cells with lower expression of EMT and *stemness* genes. Heterogenic CSC populations are common and have been observed in other tumor entities, such as breast cancer where a mix of E/M hybrid CSCs has been observed, correlating to higher *stemness* and worse overall survival 18. These mixed heterogenic states are difficult to define as specific markers for specific CSCs or CSC states do no exists, and even though a wide array of potential markers have been described in the literature, it is nearly impossible to associate some *stemness* genes with specific prognostic markers for GC. Our results show that no changes were found in E-cadherin and Vimentin between ADH and CSCs, which may be due to the fact that cancer cells are able to switch between different phenotypic states. However, low MAD2 gastrospheres from MKN45 exhibit a decrease in EMT-transcription factors such as *VIM* and *SNAI2* and many *stemness* genes and CSC-associated cell surface markers, indicating a shift in the GCSC population as a whole, shifting the balance from GCSC with high tumorigenic capacity (e.g. tumor-initiating GC *stem*-like cell) to GCSCs with low tumorigenic capacity 31.

CXCR4 is a G-protein-coupled receptor for stromal-derived-factor-1 (SDF-1) which, in its activated state, is able to modulate signalling pathways that in turn activate the expression of genes including some MMPs involved in metastasis 2. Therefore, inhibition of CXCR4 in osteosarcoma decreases invasion and MMP1 expression 32. Related to this, our data shows the same correlation in cells *in vitro*, low MAD2 in MKN45^CSCs^ decreases CXCR4 expression and MMP1 expression and activity, which correlates with a reduction in their migratory and invasive capacity *in vitro*. In addition, downregulation of MAD2 in MKN45 cells significantly reduces the growth of xenografts in immunocompromised mice, concomitant with a decrease in MMP1 and *SNAI2*. Based on this, we hypothesize, what is known in breast cancer 33, that *SNAI2* controls MMP1 expression in GCSCs. In agreement with our *in vivo* results, it is known that MAD2 levels modulate the ratio of hematopoietic *stem* cells/progenitors to engraft, repopulate and serially transplant into recipient mice 10. We believe that the experiments presented herein strongly suggest that MAD2 could be implicated in the metastasis processes of GC, as 1) it is overexpressed in most adenocarcinomas, and 2) MAD2 inhibition compromised the viability of GCSCs both *in vitro* and *in vivo*.

The M2i-1 inhibitor has been shown to disrupt the assembly of the mitotic checkpoint complex (MCC) 34, 35. Our results show that GCSC *in vitro* viability and self-renewal are significantly impacted in presence of M2i-1, and in the *in vivo* experiments, intermediate tumor growth kinetics and growth are observed. The less potent effect *in vivo* is likely due to the fact that the pharmacological effect is lost or the doses for treatment *in vivo* are not as effective as *in vitro*. Further research is needed in order to find new molecules that target MAD2 and to clarify the strong effect of this protein over GCSCs. Recent studies indicate that combined treatment with Taxol and M2i-1 increases HeLa cell death, by promoting apoptosis 36. Furthermore, MAD2 siRNA (siMAD2)-loaded on nanoparticles, has been presented as an attractive drug delivery platform for RNAi therapeutics against NSCLC 37. It would be interesting to verify if the incidence of GC tumors could also be reduced with similar MAD2-targetting therapeutic approaches and combinations.

One of our results which clearly need to be thoroughly investigated in the near future is the influence of MAD2 levels on transcriptional regulation. We have clearly shown that when MAD2 is depleted the transcriptional program of GCSCs changes; however, the effect was variable across GC cell lines. One explanation could be a change in the transcriptome during the process of differentiation of GCSCs and EMT, which could influence not only the expression of certain transcription factors and therefore the induction of repressed genes, but also microRNA expression 38. Another possibility is that since MAD2 is a flexible protein thanks to its HORMA domain, its modulation can have radically different functional outcomes beyond just mitosis, as has been recently described and may explain some of our findings 39. The module p31Commet-MAD2-BUBR1 binds the insulin receptor linking mitotic proteins to nutrient metabolism 40. Wilms' tumor-1 protein (WT1) is a transcription factor that can either activate or repress genes to regulate cell growth, apoptosis and differentiation, and it is also able to interact with MAD2 and modulate mitosis control 41. Finally, a MAD2-mediated translational regulatory mechanism promoter S-phase cyclin synthesis has been described in yeast 42. We should like to finish off with one last consideration regarding the MAD2 function outside mitosis in *stem* cells. The normal state for these cells is quiescence, in which cells are outside cell cycle, so the high levels of MAD2 suggest new biological functions to study.

**Figure 8:**
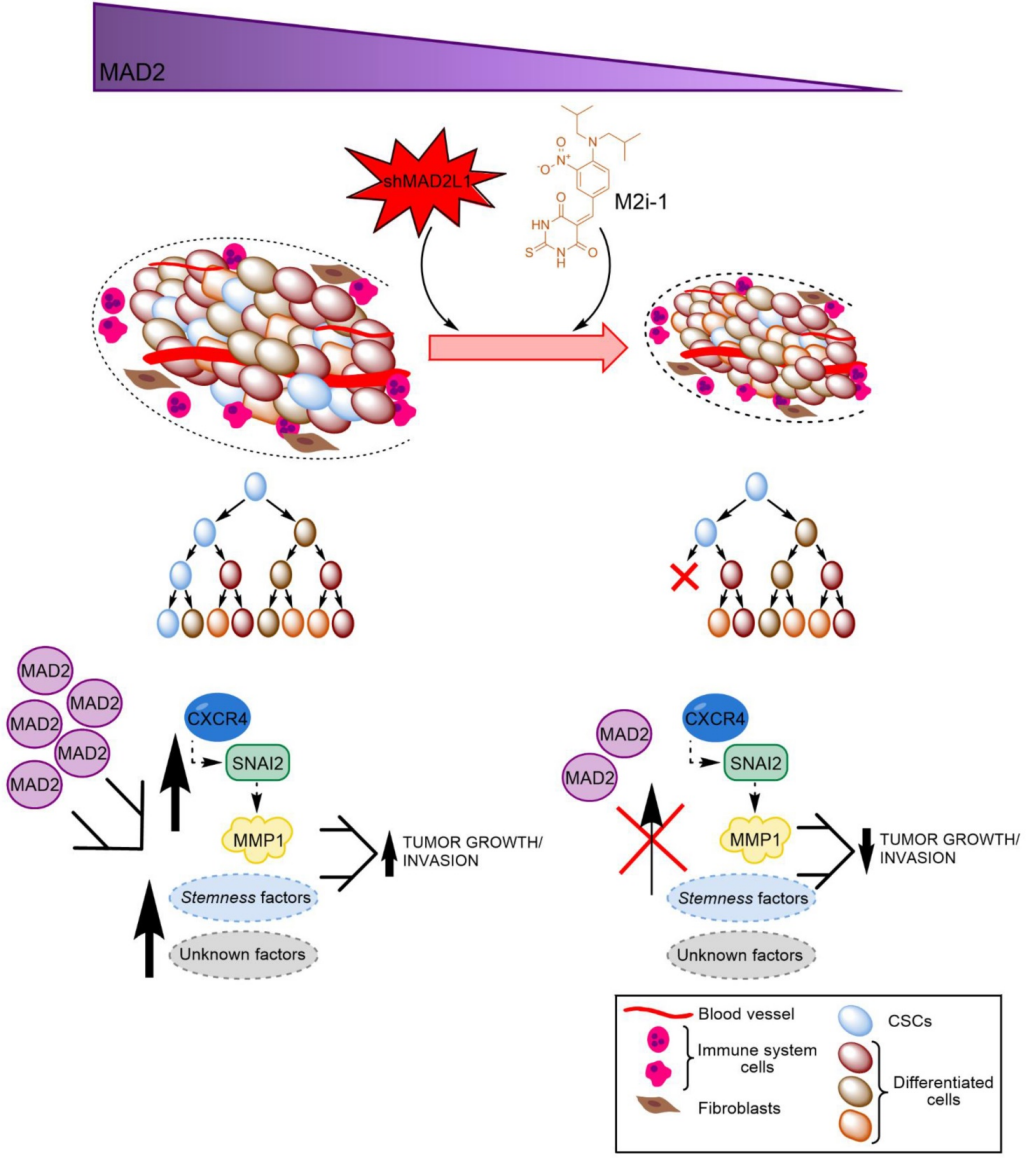
** Working model of how MAD2 downregulation promotes asymmetric division and reduces tumor growth and invasion in MKN45 cell line.** CSCs with high levels of MAD2 (left) enhances symmetric division and increases tumor growth by activating the signaling pathway CXCR4-*SNAI2*-MMP1. Targeting MAD2, with shMAD2L1 or M2i-1 inhibitor (right), could downregulate this pathway, promoting GCSC asymmetric division and lower metastasis, and as a consequence, decreasing GCSC tumorigenic capacity.

Finally, considering the aforementioned role of MAD2 in tumorigenesis, we hypothesize that GCSCs from tumors with high levels of MAD2 have an activated CXCR4-*SNAI2* signalling pathway, increasing the expression of MMP1, which translates into higher invasion and tumor growth. Targeting MAD2 could downregulate this pathway, promoting asymmetric division and lowering metastasis (Figure 8). Without a doubt, the role of MAD2 appears to extend beyond that of merely modulating mitosis, and its utility in the search for new therapeutic avenues to control cancer and CSCs should be fully exploited.

## Supplementary Material

Supplementary figures.

## Figures and Tables

**Table 1:** Primer sequences

Primer	Sequence
*CDH1* (E-cadherin)	F: CCATTCTGGGGATTCTTGGAGG
	R: GCAGCTGGCTCAAGTCAAAGTC
*VIM* (Vimentin)	F: GCTCAATGTTAAGATGGCCC
	R: CAGAGGGAGTGAATCCAG
*SOX2*	F: GCACATGAACGGCTGGGAGC
	R: GCGAGTAGGACATGCTGTAGG
*NANOG*	F: TCTCTCCTCTTCCTTCCTCC
	R: GGAAGAGTAAAGGCTGGGG
*OCT3/4*	F: GCAACCTGGAGAATTTGTTCC
	R: GACCCAGCAGCCTCAAAATC
*LGR5*	F: CCTCTGCTGGCTTTTAGGTG
	R: TGAAAGGCCTGAAAACTGCT
*LOXL2*	F: GGAGAGGACATACAATACCAAAGTG
	R: CCATGGAGAATGGCCAGTAG
*MAD2L1*	F: GCTTGTAACTACTGATCTTG
	R: GCAGATCAAATGAACAAGAA
*BUB1B*	F: TCGTGGCAATACAGCTTCAC
	R: GGTCAATAGCTCGGCTTCC
*MMP9*	F: GAGTTCCCGGAGTGAGTTGA
	R: AAAGGTGAGAAGAGAGGGCC
*MMP2*	F: GGGGTGAAAATGGAGGGAGA
	R: CCGACTCTTAAAGCTTCCGC
*MMP1*	F: CTTGCACTGAGAAAGAAGACAAAGG
	R: ACACCCCAGAACAGCAGCA
*SNAI1*	F: CTCCCTGTCAGATGAGGAC
	R: CCAGGCTGAGGTATTCCTTG
*SNAI2*	F: GGGGAGAAGCCTTTTTCTTG
	R: TCCTCATGTTTGTGCAGGAG
*ZEB1*	F: CCAGGTGTAAGCGCAGAAA
	R: CCACAATATGCAGTTTGTCTTCA
*TWIST*	F: AGGGCTCTCAGAAGAGGACC
	R: AAGGAAAAGAATAGCCGGCGT
*CDKN1A* (p21)	F: GCTGCAGGGGACAGCAGAG
	R: GCTTCCTCTTGGAGAAGATCAG
*IL6*	F: GCCAGAGCTGTGCAGATGAG
	R: CAGTGGACAGGTTTCTGACC
*GAPDH*	F: GAGAGACCCTCACTGCTG
	R: GATGGTACATGACAAGGTGG

## Abbreviations

GC: gastric cancer
CSCs: cancer *stem*-like cells
MAD2: mitotic arrest deficient-like-1 protein (gene *MAD2L1*)
SAC: spindle assembly checkpoint
EMP: epithelial-mesenchymal plasticity
EMT: epithelial-mesenchymal transition
MET: mesenchymal-epithelial transition
MMP: matrix metalloproteinase
BLM: bleomycin
PXL: paclitaxel
CDDP: cisplatin
qPCR: quantitative real-time polymerase chain reaction
RT: reverse transcription
H&E: hematoxylin & eosin
PBS: phosphate-buffered saline
PI: propidium iodide
DAPI: 4',6-diamidino-2-phenylindole
GAPDH: glyceraldehyde 3-phosphate dehydrogenase
BrdU: bromo deoxyuridine
