# Supplementary material for: Targeting MAD2 modulates stemness and tumorigenesis in human Gastric Cancer cell lines
Source: Theranostics. 2020 Jul 25;10(21):9601–18. doi: 10.7150/thno.49270 (PMC7449921; doi:10.7150/thno.49270)
Supplement: Supplementary file 1 — Supplementary figures. [file thnov10p9601s1.pdf]

Figure S1

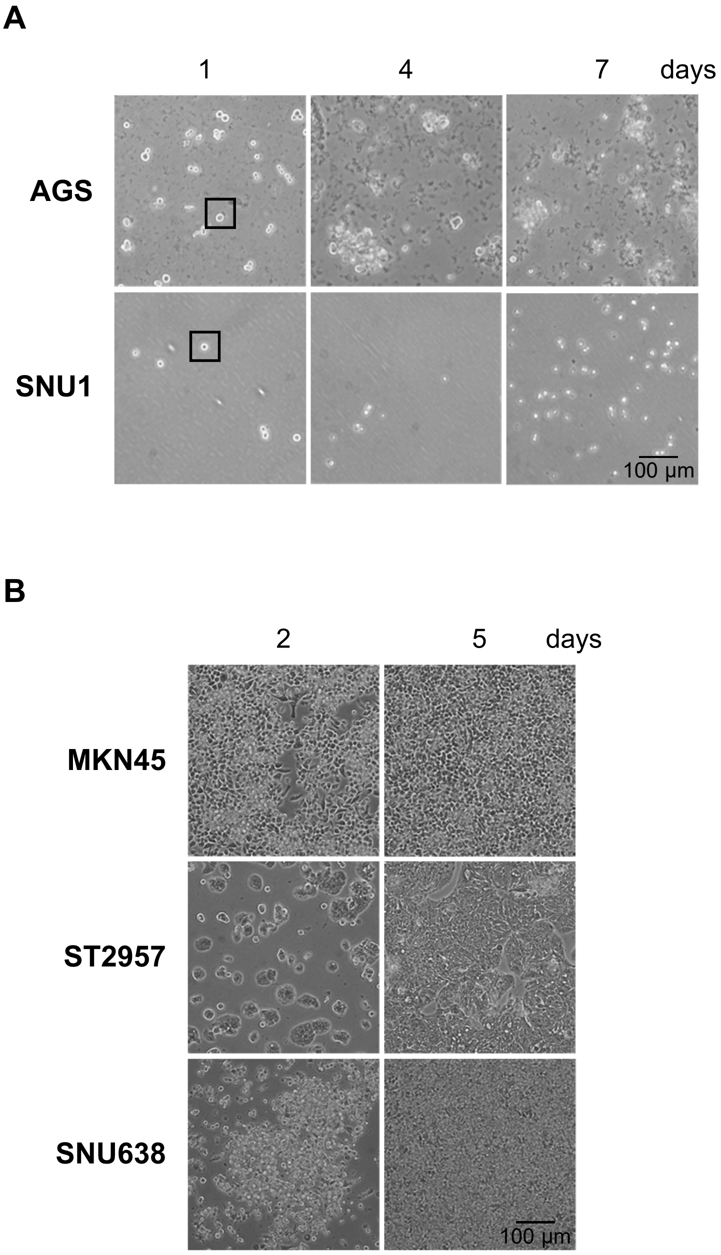

Figure S2

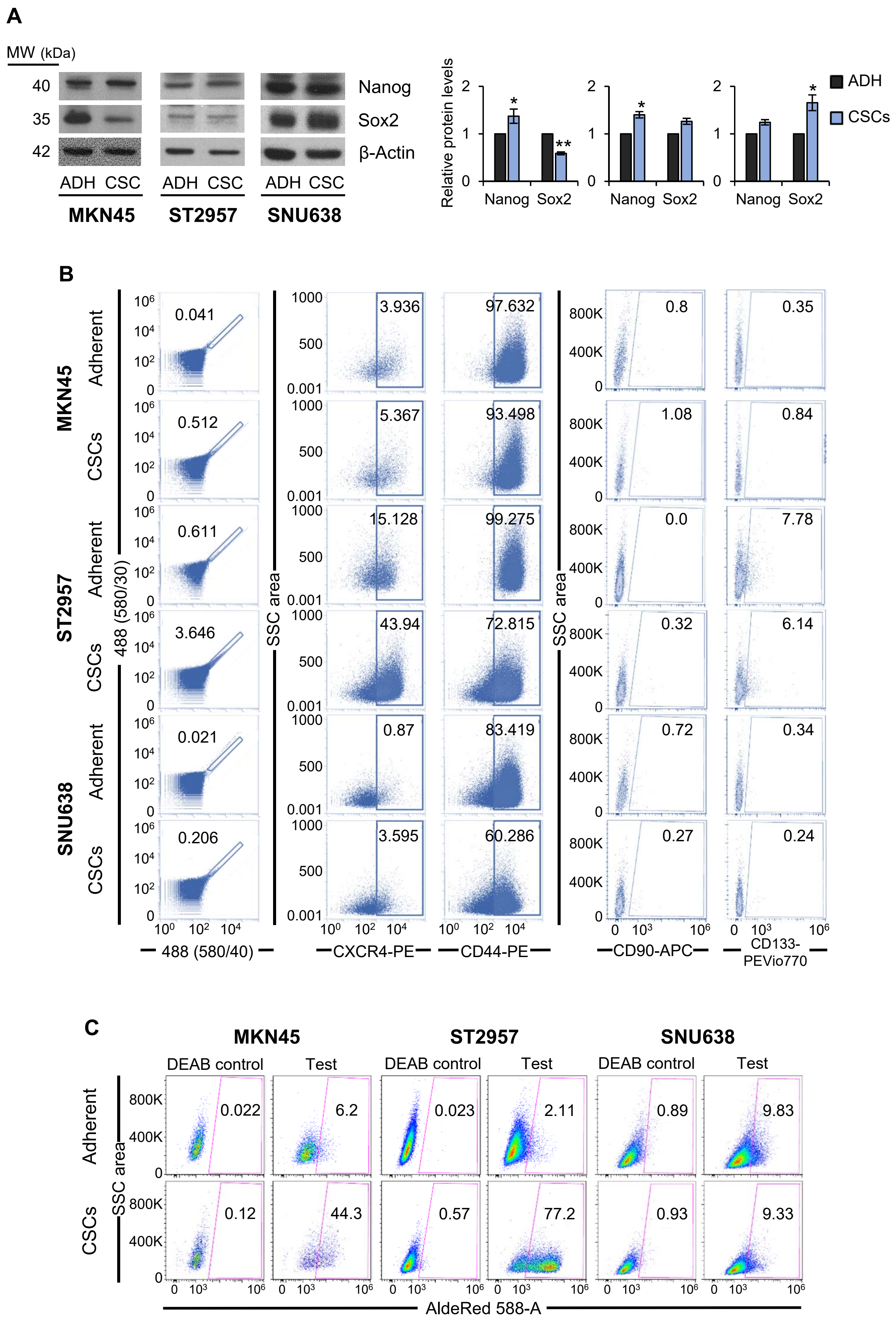

A
MW (kDa)
40
35
42
Nanog
Sox2
β-Actin
ADH CSC
ADH CSC
ADH CSC
MKN45
ST2957
SNU638
Relative protein levels
Nanog Sox2
ADH
CSCs
B
MKN45
ST2957
SNU638
Adherent
CSCs
488 (580/30)
SSC area
0.041
0.512
0.611
3.646
0.021
0.206
3.936
5.367
15.128
43.94
0.87
3.595
97.632
93.498
99.275
72.815
83.419
60.286
0.8
1.08
0.0
0.32
0.72
0.27
0.35
0.84
7.78
6.14
0.34
0.24
488 (580/40)
CXCR4-PE
CD44-PE
CD90-APC
CD133-PEVio770
C
MKN45
ST2957
SNU638
DEAB control
Test
Adherent
CSCs
SSC area
0.022
6.2
0.023
2.11
0.89
9.83
0.12
44.3
0.57
77.2
0.93
9.33
AldeRed 588-A

Figure S3

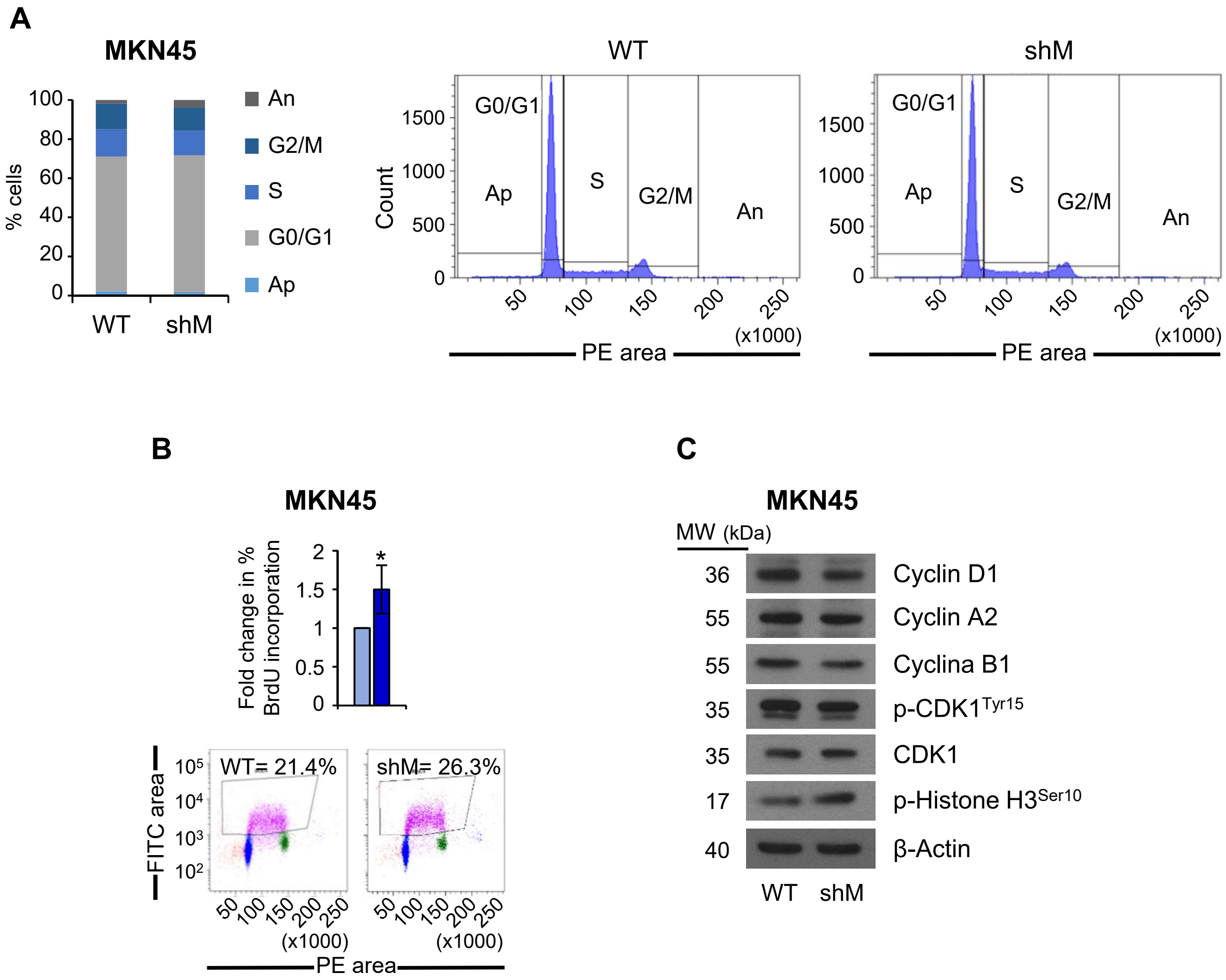

A
MKN45
% cells
100
80
60
40
20
0
WT
shM
An
G2/M
S
G0/G1
Ap
WT
shM
Count
1500
1000
500
0
G0/G1
Ap
S
G2/M
An
50
100
150
200
250
(x1000)
PE area
B
MKN45
Fold change in % BrdU incorporation
2
1.5
1
0.5
0
*
FITC area
WT= 21.4%
shM= 26.3%
PE area
C
MKN45
MW (kDa)
36
55
55
35
35
17
40
Cyclin D1
Cyclin A2
Cyclina B1
p-CDK1Tyr15
CDK1
p-Histone H3Ser10
β-Actin
WT
shM

Figure S4

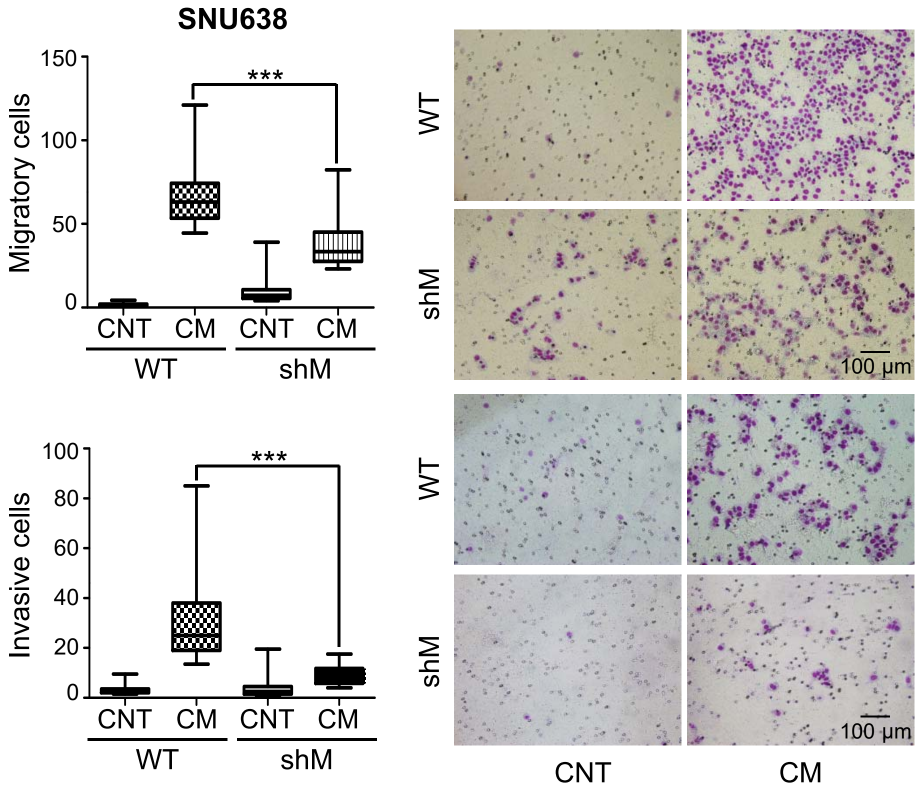

SNU638
Migratory cells
150
100
50
0
***
CNT
CM
CNT
CM
WT
shM
Invasive cells
100
80
60
40
20
0
***
CNT
CM
CNT
CM
WT
shM
WT
shM
WT
shM
100 μm
100 μm
CNT
CM

Figure S1. Isolation of GCSCs from GC cell lines. (A) Sphere formation assay of AGS and SNU1 GC cell lines. Cells were grown in ultra-low-attachment 6-well plates with DMEM/F-12 medium (3000 cells per well) containing specific supplements. Representative images were taken 1, 4 and 7 days later. Scale bar: 100 µm. **(B)** Re-differentiation of CSCs from MKN45, ST2957 and SNU638 cell lines. CSCs were grown in adherent conditions with 10% FBS medium. Representative images were taken 2 and 5 days later. Scale bar: 100 µm. The experiments were repeated two times with similar results.

Figure S2. GCSCs characterization. (A) Western blot analysis of NANOG and SOX2 in the GC cell lines MKN45, ST2957 and SNU638 cultured in adherence (ADH) or as sphere (CSCs). Right, graphs show the fold change of protein levels compared to ADH. Protein levels were normalized with β-actin. WB images are representative of the results obtained in three different experiments performed under the same conditions. The statistical significance was evaluated with two-tailed Student's t-test (*$P < 0.05$; **$P < 0.01$; ***$P < 0.001$). **(B)** Flow cytometric analysis of autofluorescence (580/30-580/40), CXCR4, CD44, CD90 and CD133 cell surface markers in the 3 GC cell lines and their CSCs. Representative plots with the percent-positive cells present within the single-cell, live and debris-free population are shown. **(C)** ALDH activity profile. The 3 GC cell lines and their CSCs were counter-stained with AldeRed to determine the frequency of ALDH-positive cells by flow cytometry. Representative plots are shown, in the presence or absence of the ALDH inhibitor (DEAB), with the percent-positive cells present within the single-cell, live and debris-free population.

Figure S3. *MAD2L1* interference does not affect proliferation in MKN45^{CSCs}. (A) Cell cycle profile of MKN45^{CSCs}. MKN45 2nd generation-enriched CSC cultures after *MAD2L1* interference (shM) or not (WT) were analyzed by flow cytometry. The graph

shows the percentage of cells in each phase of the cell cycle. An = aneuploidy (cells > 4n); G2/M = G2/mitosis; S = synthesis; G0/G1; Ap = apoptosis (cells < 2n). **(B)** BrdU incorporation analysis. MKN45^{CSCs} WT and shM were incubated with BrdU (10 µM) for 1 hour and analyzed by flow cytometry. Representative plots with the percent-positive cells present within the single-cell, live and debris-free population are shown. On top, histogram summarizing the percent of BrdU-positive cells in sphere cultures from three different experiments. Fold changes were calculated compared to WT. **(C)** Western Blot analysis for the cell cycle proteins Cyclin D1, Cyclin A2, Cyclin B1, p-CDK1^{Tyr15}, CDK1 and p-Histone H3^{Ser10} in MKN45^{CSCs} WT and shM. β-actin was used as a loading control. The experiments were repeated three times with similar results. Statistical significance was evaluated with two-tailed Student's t-test (*$P < 0.05$).

Figure S4. MAD2 levels regulate migration and invasion in SNU638^{CSCs}. Transwell migration (top) and invasion (bottom) assay in SNU638^{CSCs} after MAD2 downregulation. Graphs show the quantification of stained migratory and invasive cells, respectively, from CSCs WT and shM using the transwell assay, without and with 20% serum-free conditioned medium from M2-differentiated human macrophages (CM) after 48 h. Representative photographs of the experiment on the right. Scale bar: 100 µm. Three independent experiments were performed with similar results. The statistical significance was evaluated with one-way ANOVA, post hoc comparisons, Bonferroni´s test (***$P < 0.001$).
